# The potential role of Alu Y in the development of resistance to SN38 (Irinotecan) or oxaliplatin in colorectal cancer

**DOI:** 10.1186/s12864-015-1552-y

**Published:** 2015-05-22

**Authors:** Xue Lin, Jan Stenvang, Mads Heilskov Rasmussen, Shida Zhu, Niels Frank Jensen, Line S Tarpgaard, Guangxia Yang, Kirstine Belling, Claus Lindbjerg Andersen, Jian Li, Lars Bolund, Nils Brünner

**Affiliations:** Department of Biomedicine, University of Aarhus, the Bartholin Building, DK-8000 Aarhus C, Denmark; Department of Veterinary Disease Biology, Section of Molecular Disease Biology, Faculty of Health and Medical Sciences, Copenhagen University, Strandboulevarden 49, Copenhagen, Denmark; Department of Molecular Medicine, Aarhus University Hospital, Brendstrupgårdsvej 100, DK-8200 Aarhus N, Denmark; BGI (Beijing Genomics Institute), Shenzhen, 518083 China; Department of Oncology, Odense University Hospital, Sdr. Boulevard 29, DK-5000 Odense C, Denmark; Center for Biological Sequence Analysis, Department of Systems Biology, Technical University of Denmark, 2800 Lyngby, Denmark; The Key Laboratory of Developmental Genes and Human Disease, Ministry of Education, Institute of Life Sciences, Southeast University, Nanjing, 210096 China

**Keywords:** Alu, DNA methylation, Colorectal cancer, SN38 and oxaliplatin resistance, Diversity, Cell fate dynamics

## Abstract

**Background:**

Irinotecan (SN38) and oxaliplatin are chemotherapeutic agents used in the treatment of colorectal cancer. However, the frequent development of resistance to these drugs represents a considerable challenge in the clinic. Alus as retrotransposons comprise 11% of the human genome. Genomic toxicity induced by carcinogens or drugs can reactivate Alus by altering DNA methylation. Whether or not reactivation of Alus occurs in SN38 and oxaliplatin resistance remains unknown.

**Results:**

We applied reduced representation bisulfite sequencing (RRBS) to investigate the DNA methylome in SN38 or oxaliplatin resistant colorectal cancer cell line models. Moreover, we extended the RRBS analysis to tumor tissue from 14 patients with colorectal cancer who either did or did not benefit from capecitabine + oxaliplatin treatment. For the clinical samples, we applied a concept of ‘DNA methylation entropy’ to estimate the diversity of DNA methylation states of the identified resistance phenotype-associated methylation loci observed in the cell line models. We identified different loci being characteristic for the different resistant cell lines. Interestingly, 53% of the identified loci were Alu sequences- especially the Alu Y subfamily. Furthermore, we identified an enrichment of Alu Y sequences that likely results from increased integration of new copies of Alu Y sequence in the drug-resistant cell lines. In the clinical samples, *SOX1* and other *SOX* gene family members were shown to display variable DNA methylation states in their gene regions. The Alu Y sequences showed remarkable variation in DNA methylation states across the clinical samples.

**Conclusion:**

Our findings imply a crucial role of Alu Y in colorectal cancer drug resistance. Our study underscores the complexity of colorectal cancer aggravated by mobility of Alu elements and stresses the importance of personalized strategies, using a systematic and dynamic view, for effective cancer therapy.

**Electronic supplementary material:**

The online version of this article (doi:10.1186/s12864-015-1552-y) contains supplementary material, which is available to authorized users.

## Background

Colorectal cancer is a common and often lethal disease [1]. FOLFIRI (folinic acid, 5-fluorouracil and irinotecan) [2], FOLFOX (folinic acid, 5-fluorouracil and oxaliplatin) [3] and XELOX (capecitabine and oxaliplatin) [4] are commonly used chemotherapeutic combinations used to treat colorectal cancer. However, a considerable subpopulation of patients will experience disease recurrence due to acquired resistance to treatment. The molecular mechanisms underlying acquired resistance to these drugs remain elusive.

Cancer cells usually harbour numerous genomic and epigenomic aberrations, thereby presenting high diversities of genotypes and phenotypes as well as cell fate dynamics. For somatic cells, cell fate is well defined and stably maintained by epigenetic mechanisms. DNA methylation is a long-term stable epigenetic mechanism. Additionally, DNA methylation represses the activity of mobile genetic elements and maintains genome integrity.

The concept ‘eukaryotic genomes are dynamic’ has been well accepted [5] since mobile genetic elements were first discovered in maize [6]. One remarkable feature in the human genome is that the DNA consists of at least 45% mobile genetic elements, including short interspersed nuclear elements (SINEs), long interspersed nuclear elements (LINEs) and long terminal repeats (LTRs) [7]. LINE-1 (L1) is a predominant member of LINEs and Alu is the largest family of SINEs in the human genome [7]. Moreover, L1s are the only autonomous retrotransposable element in the human genome. Alus are non-autonomous retrotransposable elements, which depend on an L1 coded protein ORP2 (endonuclease and reverse transcriptase) to mediate their mobility. Alus are primate-specific sequences sharing a typical 282-nucleotide consensus sequence and a characteristic structure [8]. There are more than one million Alu family members, constituting 11% of human DNA [8]. The members are ubiquitously dispersed throughout the genome but preferentially overrepresented in GC-rich and high gene density regions [8]. It is thought that about 75% of the total number of genes in the genome are associated with Alus [9]. The presence of Alu sequences is strongly correlated with multifractality in human genome sequences [10]. Alu elements are also associated with more than 25% of all the simple repetitive sequences in primate genomes, including microsatellites [11]. It is reported that Alu and L1 initiate the spread of CpG methylation, and the length of CpG islands is associated with the distribution of Alu and L1 retrotransposons [12]. Moreover, Alu elements are supposed to act as global modifiers of gene expression through changes in their own methylation state [8]. It is estimated that there are about 80-100 active L1s and 2000-3000 active Alus in the human genome per individual [13,14]. Mobilization of Alus mainly occurs during the production of gametes or at early stages of embryo development [5]. In contrast to germ line retrotransposition, the activity of Alus and other mobile genetic elements in somatic cells is mostly silenced by DNA methylation and post-transcriptional mechanisms mediated by piwi-interacting RNAs, siRNAs, miRNAs and *AID/APOBEC* gene family members [5,15-18]. However, genomic toxicity induced by carcinogens or drugs can reactivate Alus by altering DNA methylation [19]. Accordingly, Alu and L1 have been shown to display DNA methylation alterations in colorectal cancers compared with matched normal tissues [20,21]. Additionally, Alu elements pose the largest transposon-based mutagenic threat to the human genome [14]. A recent study, which intensively sequenced 43 cancer and matched germ line genomes, revealed that colorectal cancers and other cancers of epithelial cell origin show activity of somatic L1 and Alu transpositions [22].

Among Alu sequences, the Alu Y subfamily is the youngest Alu sequence [8] with an evolutionary age of ~15-20 million years (Mya) [23]. Even though the copy number of Alu Y (~125,000 copies) is less than that of Alu S (550,000 copies, at evolutionary age ~40-50 Mya [23]) and Alu J (~160,000 copies, at evolutionary age ~55 Mya [23]), the Alu Y subfamily harbours the largest number of functionally intact Alu core elements that are more active than the older Alus [14,24]. Activation of Alus can have many important biological consequences: Alus can reshuffle the genome, generating transposon-mediated mutagenesis [25], inducing genomic instability [26], and increasing recombination between elements [8], thereby contributing to genetic population diversity [8,27] as well as to heterogeneity in tumorigenesis. Alus can also remodel the epigenome and alter gene expression patterns by changing epigenetic marks of neighbouring genes at new insertion sites, introducing ectopic promoters of transcription factor binding sites, and generating novel alternative splicing. Integration of Alu sequences and subsequent remodelling of DNA methylation might lead to epigenetic reprogramming [28] as well as pluripotency induction and maintenance by A-to-I RNA editing of Alu sequences [29]. Whether Alu retrotransposition occurs during chemotherapy with SN38 or oxaliplatin, and thereby plays a potential role in the development of chemotherapy resistance, remains unknown.

We hypothesized that development of drug resistance in colorectal cancer follows a linear step-wise progressive model and in the present study, we applied reduced representation bisulfite sequencing (RRBS) assay to analyse the DNA methylome from 3 established SN38-resistant and 3 established oxaliplatin-resistant human colorectal cancer cell line models [30]. Our results indicate a potential role of Alu elements, especially the Alu Y subfamily, in the resistance to SN38 and oxaliplatin. To validate the findings from the cell line models, we extended our RRBS analysis to 14 clinical colorectal cancer samples. Based on the analyses of the cell lines and clinical samples, we have attempted to delineate the influence of altered DNA methylation on activation of retrotransposons as a model for colorectal cancer chemotherapy resistance.

## Results

### Global methylome and non-CpG methylation in the cell line models and clinical samples

We applied the QDMR software [31] with a concept of ‘DNA methylation entropy’ adopted from the ‘Shannon entropy’ [32], to identify differentially methylated cytosines (DMCs) by estimating variability of DNA methylation states between all colorectal cancer cells and clinical samples. DMCs in all samples in the context of CpG as well as CHG and CHH (where H means A, T or C), were identified. Unsupervised clustering using DMCs in the context of CpG, CHG and CHH were performed (Additional file 1: Figure S1A, 1B and 1C). The drug-resistant cell lines clustered with their parental cell origin in the dendrogram representing the methylome profiles. This clustering represents the phenomenon “somatic memory” and is in accord with data from gene expression profiles from the cell lines [30]. Thus, the somatic memory leads to clustering of the resistant and parental cells rather than clustering according to specific drug-resistance. Also, all cell lines merged in a big cluster separated from the clinical samples in unsupervised clustering, suggesting that the colorectal cancer cell lines might show similar features of DNA methylome, whereas sporadic clinical samples show high diversity between individual methylomes. In the cell line studies, analyses were performed after chemotherapy-induced resistance while in the clinical cancers the samples for analyses were obtained prior to any chemotherapy. This could also explain separation between the cell line samples and clinical samples in the unsupervised cluster analysis. An additional factor distinguishing the clinical samples from the cell lines is the clinical samples contain a mixture of cells including cancer cells, stromal cells and endothelial cells whereas the cell lines are much less heterogeneous.

There were a certain number of non-CpG cytosine (CHH and CHG) methylations in all colorectal cancer cell lines and sporadic colorectal cancer samples. Clustering based on non-CpG cytosine methylation data was largely consistent with that based on CpG cytosine methylation data (Additional file 1: Figure S1B and 1C). This suggests that both CpG methylation and non-CpG methylation reflect the general somatic memory of DNA methylation modification. From the identified DMCs, we selected the non-CpG cytosine loci (CHH and CHG) shared by both the cell line models and the 14 clinical samples with a methylation level of at least 50% or higher in every sample. There were totals of 19 and 29 cytosine loci identified, in CHG and CHH formats respectively. Among the CHG methylated cytosine loci, 12 loci were located in gene bodies (exon, intron, promoter and TSS (transcription start site)) and 7 loci in intergenic regions. Among the CHH methylated cytosine loci, 15 loci were located in coding genes, 1 locus in a microRNA gene, and 13 in intergenic regions. For the CHG and CHH loci located in intergenic regions, most of them were in repeat sequences. For example, there were 3 and 6 loci harbouring in Alu elements in CHG and CHH formats, respectively. Interestingly, 2 of these (out of 3) and 5 (out of 6), respectively, belonged to the Alu Y subfamily. The information concerning the identified CHG and CHH methylated loci is available in the Additional file 2: Table S1A and 1B, respectively.

**Figure 1:**
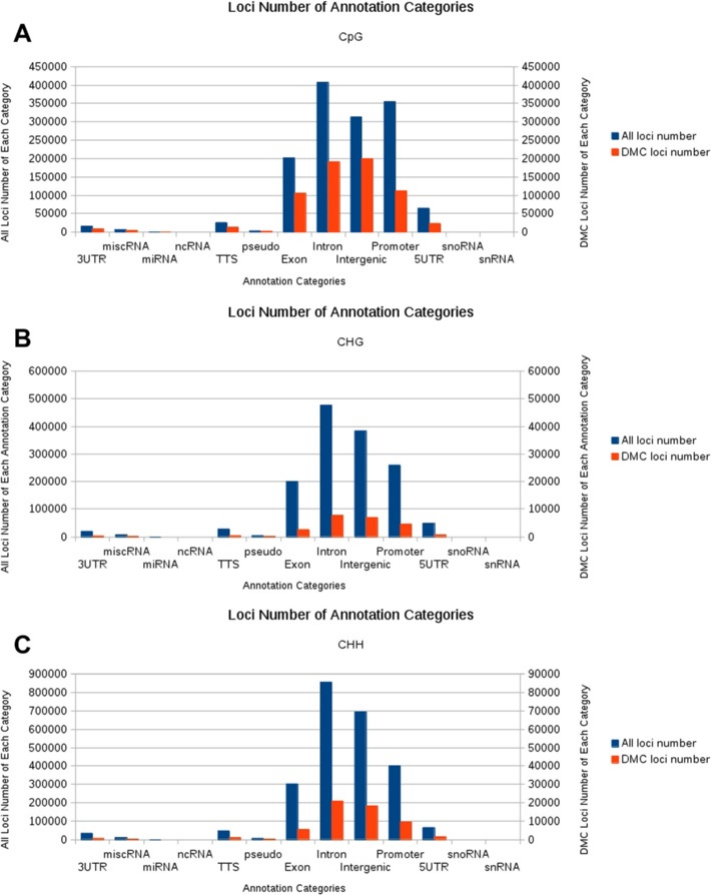
The distribution of differentially methylated cytosines (DMCs) in different genomic components. **A** shows the distribution of total cytosines (blue bars) and DMCs (red bars) in the context of CpG in different components of the human genome. **B** and **C** show the distribution of total cytosines (blue bars) and DMCs (red bars) in the context of CHH and CHG in different components of the human genome, respectively. The height of the left vertical axis indicates the number of total cytosines in the context of CpG **(A)**, CHG **(B)** and CHH **(C)**, respectively. The height of the right vertical axis indicates the number of the DMCs in the context of CpG **(A)**, CHG **(B)** and CHH **(C)** for the given genomic components, respectively.

Furthermore, we investigated the distribution of the DMCs in different genomic components. The DMCs (composed of CpG, CHH and CHG) were mainly located in intergenic and intronic regions. Notably, even though the majority of CpG loci harboured more frequently in introns and promoters than in intergenic regions, the largest number of DMCs in CpG context were found in intergenic regions (Figure 1A). Compared with the DMCs in a CpG context, DMCs in non-CpG contexts were less frequent, residing mainly in introns, intergenic regions and promoters (Figure 1B and Figure 1C). This result suggests that CpG DMCs contribute predominantly to the DNA methylation difference in intergenic regions.

### The cytosine loci uniquely presented in the different drug-sensitive or drug-resistant phenotypes enrich Alu elements

**Figure 2:**
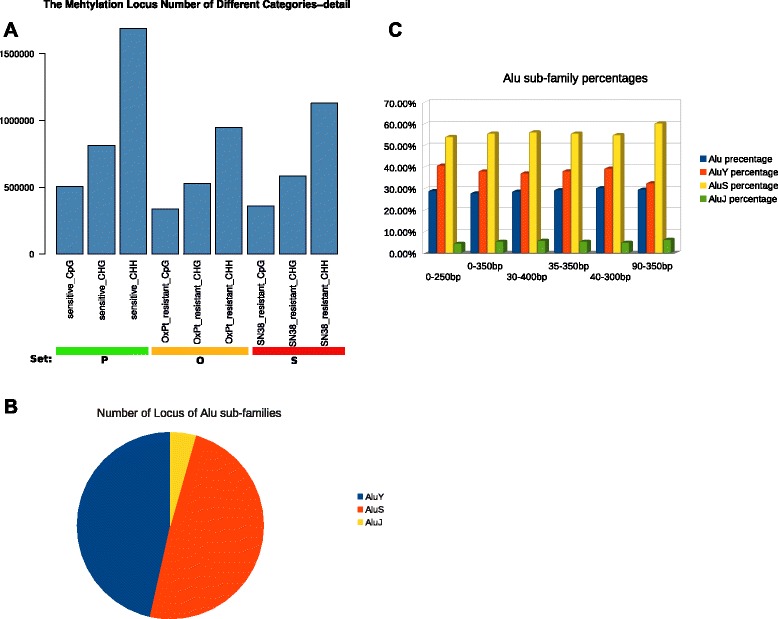
Alu Y subfamily enrichment. **A**: the number of selected loci in the sets *P*, *O* and *S* (see text). **B**: the percentage of the Alu Y subfamily out of the identified Alu elements in set *E*. **C**: the percentage of Alu elements in all cytosine loci and the percentage of the Alu Y subfamily in the identified Alu elements in either sliding windows of fixed size or extended size in RRBS *in-silico* simulation.

By analysing RRBS data for the colorectal cancer cell line models, we identified the loci unique to the three parental cell lines (cell line A (HCT-116 parental), D (HT-29 parental) and G (LoVo parental)) and defined a set *P*, which contains only these parental cell line loci (including the three formats of CpG, CHG and CHH). We then identified loci unique to the three OxPt-resistant sub-lines (cell line B (HCT-116 OxPt resistant), E (HT-29 OxPt resistant) and H (LoVo OxPt resistant)) as uniquely representing OxPt-resistant DNA methylation features and defined a set *O*, which contains only these OxPt-resistant sub-line loci (including the three formats of CpG, CHG and CHH). Finally, we identified the loci unique to the three SN38-resistant sub-lines (cell line C (HCT-116 SN38 resistant), F (HT-29 SN38 resistant) and I (LoVo SN38 resistant)) as uniquely representing SN38-resistant DNA methylation features and defined a set *S*, which contains only these SN38-resistant sub-line loci (including the three formats of CpG, CHG and CHH). The numbers of the identified loci in sets *P*, *O* and *S* are shown in Figure 2A. The definitions of the different sets in this study are available in the Additional file 3: Table S2. Taking the identified loci in the context of CpG as an example, there were 505,147, 337,242 and 359,770 cytosine loci identified as uniquely representing drug-sensitive DNA methylation features in the parental (*P*), OxPt-resistant (*O*), and SN38-resistant (*S*) cell-lines respectively.

We found that sets *P*, *O* and *S* are highly enriched in Alu elements, which accounted for 48.8%, 60.1% and 53.3% of all identified cytosine loci, respectively*.* Notably, many identified Alu elements belong to the youngest Alu subfamily – Alu Y – accounting for 32.1%, 35.8% and 34.3% in the identified Alu elements in the set *P*, *O* and *S*, respectively. Subsequently, we performed RRBS analysis for the 14 clinical samples, and then selected the cytosine loci commonly found in these clinical samples, defined as set *C*. We further selected the cytosine loci by only keeping the loci that were commonly found in both set *C* and the *united* set of *P*, *O* and *S* and defined a new set *E*, contains only the commonly shared cytosine loci. We identified 48,944 loci in set *E*. Information about these set *E* cytosine loci is available in the Additional file 4: Table S3. Notably, the percentage of Alu elements among the identified loci in set *E* was 53.4%, and the percentage of the Alu Y subfamily in all identified Alu elements in the set *E* was increased to 46.3% (Figure 2B). Subsequently, we performed DMCs analysis for the identified loci and found that the Alu Y subfamily accounted for 48.4% of the identified DMCs Alu loci.

To exclude the possibility that the enriched Alu Y sequences came from a possible bias of the RRBS technique, we performed an *in-silico* simulation. We used the same reference genome (GRCh37) that was used for RRBS mapping as the virtual test genome, subjecting it to *MspI* digestion and recovery of the resulting DNA from the gel selection by keeping the proper size of the digested DNA fragments in the *in-silico* stimulation. According to our experimental protocol of RRBS library generation, there was a maximum of about 28% sequences from Alu repeats in the *in-silico* stimulation, of which 39.5% belonged to the Alu Y subfamily. Furthermore, we compared the number of Alu Y loci in the data set *P*, *O*, *S* and the number of Alu Y loci in the simulated data set, which demonstrated that the Alu Y enrichment in the set of *P*, *O*, *S* was statistically significant (Fisher’s exact test, p-value < 2.2e-16).

In consideration of the potential variance of cutting sections of agarose gel from the smear of the digested genomic DNA, we applied a sliding window by moving a fixed size selection section from the simulated smear of the digested genomic DNA in both directions (towards smaller selection size or bigger selection size) and we also extended the selected section by extending additional 50 bp towards the bigger size or towards the smaller size. Neither sliding the fixed size selection section nor extending selection size showed significant variance in the amount of Alu sequences (Figure 2C). We could not further simulate the alignment because it is hard to estimate potential DNA methylation state for the simulated cytosines. Since Alus are repetitive sequences, alignment of simulated digested genomic DNA to the human reference genome could lead to further decrease in the proportion of Alu sequences. This is due to problems with low mapping quality, which results from repetitive sequences mapping to multiple locations in the human genome reference and/or the low priority for annotation (the priority order is exon, intron, promoter, intergenic region, and finally, repetitive).

Moreover, we calculated the percentage of the Alu subfamilies (Alu Y, Alu J and Alu S) in the selected Alu elements according to the use of sliding selection window and extending selection windows with different size in the simulations. The percentages of the Alu subfamilies were generally consistent in all simulations (Figure 2C). We also calculated the variance in the range of the percentages of Alu elements and the Alu Y subfamily in the above simulations and compared these variances to the variance of both the percentage of Alu elements and the Alu Y subfamily among all the RRBS data, including all colorectal cancer cell models and the 14 clinical colorectal cancer samples. Clearly, the variance of the Alu Y subfamily in all the RRBS samples was much higher than that in the simulations, which indicates that bias from RRBS technology cannot explain the observed Alu Y enrichment in the sets *P*, *O* and *S*. The information of variance in all the RRBS samples and variance in the simulations are available in Additional file 5: Table S4.

**Figure 3:**
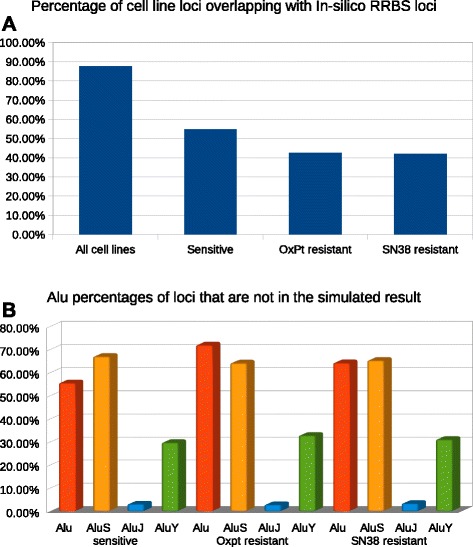
**A** shows the percentage of loci overlapping with the simulation in sets *A*, *P*, *O* and *S*. **B** shows the percentage of Alu elements, and Alu subfamilies in sets *P*, *O* and *S*, which are not overlapping with the simulation.

Subsequently, we compared the identified loci in the sets *P*, *O* and *S* with the loci in the RRBS simulation (selection section window size is 40-300 bp), respectively. We found that 54.7%, 42.4% and 42.1% loci in the sets *P*, *O* and *S* overlapped with the loci in the simulation (Figure 3A). Moreover, we identified the common loci shared by all nine colorectal cancer cell lines and defined a new set *A*. When we compared the loci in the set *A*, we found that 87.7% of the loci in set *A* overlapped with the loci in the simulation (Figure 3A). A small portion of the overlapping loci reflects the difference of the genomes of the nine cell lines from the human genome reference used in the simulation. This difference might reflect that the colorectal cancer cell lines harbour a certain number of genomic aberrations and these genomic aberrations could lead to some of the difference between the sequenced genomic loci and the loci in the simulation. Notably, set *P* from the three parental cell lines showed a lower proportion of overlapping loci with the simulation compared with set *A*, suggesting set *P* contained more loci related to genomic aberrations in individual cell lines than set *A*. Interestingly, set *P* showed higher portion of overlapping loci with the simulation than sets *O* or *S*, suggesting that the genomes of OxPt-resistant and SN38-resistant cell lines had larger extents of difference from the human genome reference than the genomes of their parental cell lines. This implies that changes in genomic structure in drug-resistant cell lines might occur during drug treatment. We further analysed the constitution of the non-overlapping loci in sets *P*, *O* and *S*. As in the previous analyses, a considerable amount of Alu elements contributed to the proportion of the non-overlapping loci in sets *P*, *O* and *S*. More impressively, the percentages of Alu elements in sets *O* and *S* were increased compared with that in set *P*. Among Alu subfamily members, we further confirmed that the Alu Y subfamily mainly contributed to the increased proportion of Alu elements in the total non-overlapping loci in the drug-resistant cell lines (Figure 3B). Thus, based on our observation and analysis, we found a correlation between the drug-resistant phenotypes and the increment of Alu Y elements.

Alu sequences can be activated and propagate into new loci of the human genome triggered by genotoxic stress [5,33]. Especially, Alu Y sequences are the biggest active subfamily of Alu elements in the human genome. The most likely explanation of our observation in the cell lines is that Alu Y elements were reactivated and spread their copies in the genome when triggered by genotoxic stress of OxPt or SN38. In the RRBS library generation, *MspI* digested the genomic DNA of the drug-resistant cell line that carried many insertions of Alus, mainly Alu Y elements. The newly inserted Alus in the drug-resistant cell lines will change the constitution of digested genomic fragments. When the digested genomic DNA underwent gel selection, the portion of the digested DNA that can be finally selected by the selection section window (40-300 bp) will be changed. Consequently, the sequenced part of the genome in the RRBS libraries from the parental cell lines and the drug resistant cell lines will be different. Taken together, the results from RRBS might reflect Alu Y subfamily retrotransposition in the drug-resistant cell lines.

**Figure 4:**
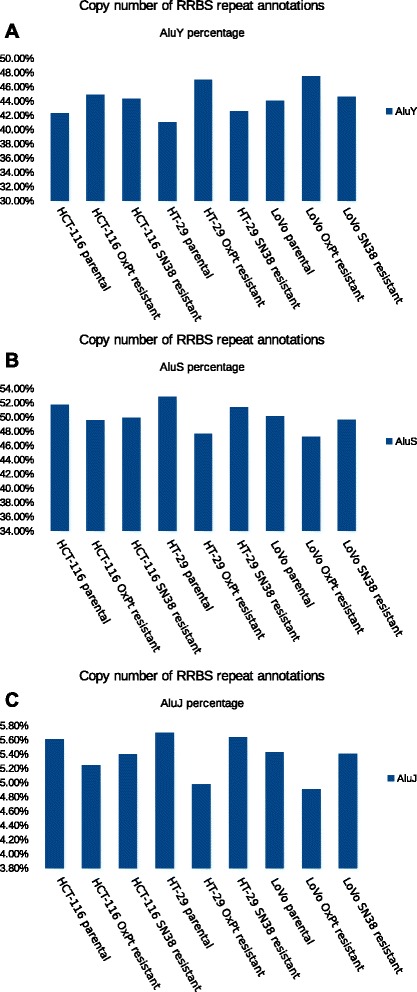
The percentage of Alu subfamilies in all colorectal cancer cell lines. **A** shows that the OxPt and SN38-resistant cell lines consistently show higher percentage of the Alu Y subfamily than their parental cell lines. **B** and **C** show the percentages of Alu J and Alu S subfamilies in the cell line models, respectively.

To further validate our findings, we compared RRBS reads that can be uniquely mapped to the human repeat sequence between the drug-resistant cell lines and their parental cell lines. We made a linear normalization of the RRBS data across all the cell lines by making the total amount of mapped RRBS reads of each sample equal. Then we extracted the reads annotated as Alu sequence and compared the number of the reads uniquely mapped to Alu sequences between the drug-resistant cell lines and their parental cell lines. In general, all OxPt and SN38-resistant cell lines consistently show higher number of reads from Alu Y subfamilies than that in their parental cell line. There is only one exception. The SN38-resistant HT-29 cell line showed almost equal number of the reads to its parental cell line. We transformed the read number into log2 format to make the distribution of read number fit a normal distribution. Then we performed a paired t-test, and the statistical result showed that the number of Alu Y reads in the drug-resistant cell lines was significantly higher than that in their matched parental cell lines (p-value = 0.0039 for the SN38 resistant cells vs. the parental cells; p-value = 2.13 × 10^−12^ for the OxPt resistant cells vs. the parental cells, respectively). Moreover, we calculated the percentage of the reads of Alu subfamily members (Alu Y, Alu J and Alu S) for all the cell line samples. The percentage of the Alu Y subfamily in the OxPt and SN38-resistant cell lines was higher than that in the parental cell lines (Figure 4A). The percentages of Alu J and Alu S subfamilies in all cell lines are shown in Figure 4B and Figure 4C, respectively.

### Identifying flanking sequence motif of Alu sequences

**Figure 5:**
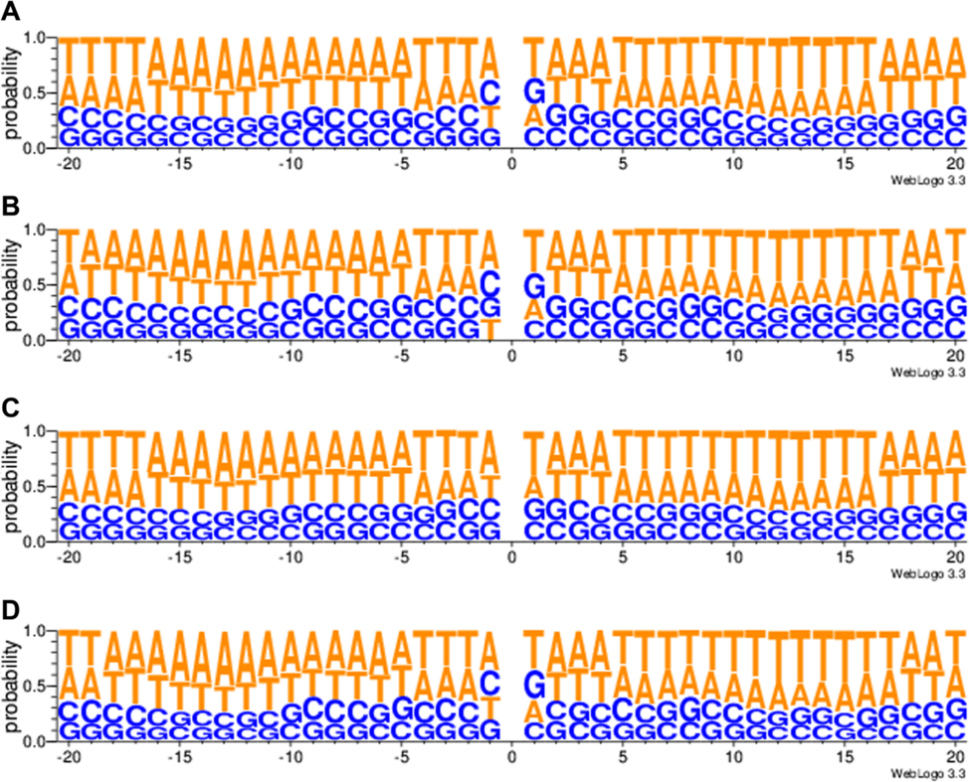
The motif of the flanking sequence of Alu elements. **A** shows the motif of the flanking sequence of the identified Alu elements in all the RRBS samples; **B** shows the motif of the flanking sequence of the identified Alu elements in set *E*; **C** shows the motif of the flanking sequence of all Alu elements in the human genome reference. **D** shows the motif of the flanking sequence of identified Alu Y subfamily in set *E*.

We applied WebLogo 3.3 [34] to extract flanking sequence (up- and down-stream 20 bp) motif from the Alu elements shared by all the RRBS samples, including both the cell lines and the clinical samples in our study. We identified a symmetric sequence in both flanking sequences of the Alu elements (Figure 5A). Furthermore, we extracted flanking sequence motifs from the identified Alu elements in set *E*, which presented the Alu elements shared by the 14 clinical samples and the united sets of *P*, *O* and *S* (Figure 5B)*.* To see whether the above Alu elements had unique features (motif sequences differing from the other Alu elements in the human genome), we extracted the flanking sequence motifs for all Alu sequences in the human genome reference (Figure 5C). Interestingly and in general, the flanking sequence of the Alus identified in all the RRBS samples and the Alus identified in set *E* both showed highly similar motif sequence to that of all Alus in the human genome reference, which is also consistent with the typical Alu target site duplication (TSD) sequence. Alu insertion depends on L1-coded ORP2, and the target site sequence of ORP2 for insertion is typically ‘TTAAAA’ [19]. Our results showed that the identified Alu sequences, either in all the RRBS samples or in set *E*, typically rely on L1-coded ORP2 for their insertion, which is in accordance with other observations [19]. Additionally, the ranking of the first flanking sequence (on the left flanking sequence) of the Alus in all the RRBS samples from the top to the bottom was A, C, T and G, according to the probability scale. Moreover, according to the probability scale, the ranking of the first flanking sequence of the Alus in set *E*, from the top to the bottom, was A, C, G and T. By contrast, the ranking of the first flanking sequence of the Alus in the whole human genome was A, T, C and G. We also extracted the flanking sequence motifs for the Alu Y subfamily in set *E*. The order of ranking the first flanking sequence from the top to the bottom was A, C, T and G, according to the probability scale (Figure 5D). These observations suggest that the additional Alu elements and Alu Y subfamily elements in this study preferably locate in GC-rich region. Since GC-rich regions are also gene-dense regions, our result implies their activity might have an effect on gene function.

**Figure 6:**
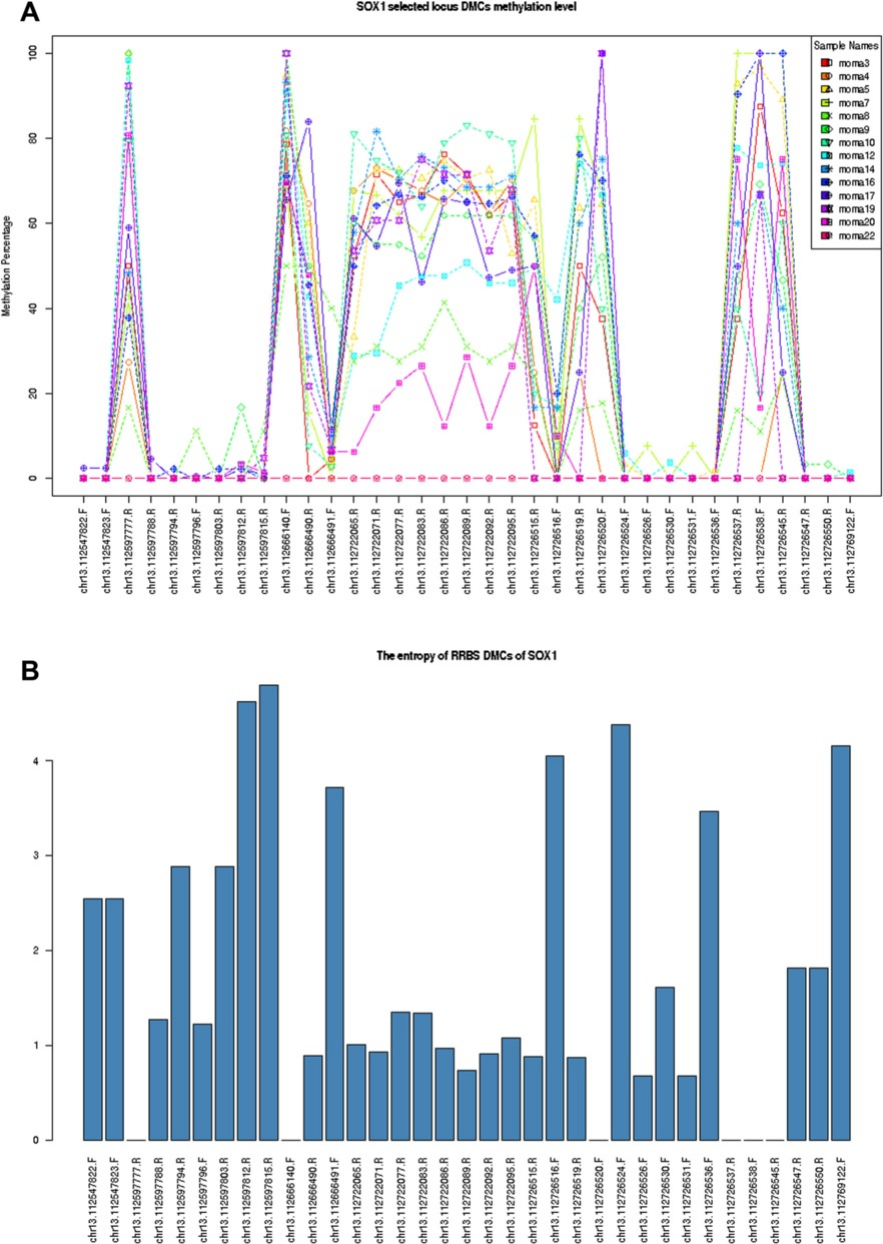
The DNA methylation states **(A)** and the diversity of DNA methylation (entropy) **(B)** of the *SOX1* gene for the 14 clinical samples.

### The identified cytosine loci in Set E highlight the high diversity of DNA methylation in SOX1 in the clinical samples

We identified 48,944 loci in set *E*, which were related to a total of 5,816 genes. Thus, many genes harbour more than one identified cytosine locus. For example, the *TSPYL2* gene harbours 90 cytosine loci, ranking first among the identified total of 5,816 genes. This gene encodes a testis specific protein, Y-encoded-like 2 (*TSPYL2*), which is a nucleosome assembly protein and plays a role in chromatin remodelling to determine gene expression, cell proliferation, and terminal differentiation [35]. Notably, *SOX1* harbours 35 of the identified cytosine loci, ranking fifteenth among the 5,816 genes. In addition to *SOX1*, other *SOX* gene members including *SOX2*, *SOX3*, *SOX4*, *SOX6*, *SOX8*, *SOX11*, *SOX14*, *SOX18*, *SOX21* and *SOX30* have been identified to harbour at least one identified cytosine locus. Thus there was a clear enrichment of the *SOX* gene family in this data set. The gene list and the number of harboured loci are available in Additional file 6: Table S5.

We extracted the DNA methylation information of the 14 clinical samples on the basis of the cytosine loci in set *E*. Furthermore, we estimated the diversity of DNA methylation states across the 14 clinical samples by measuring the DNA methylation entropy using QDMR [31]. Interestingly, many cytosine loci located in the *SOX1* gene region showed high diversity of DNA methylation states across all the 14 clinical samples (Figure 6A and B). The cytosine loci harboured in the *TSPYL2* gene and their DNA methylation level and entropy are shown in Additional file 7: Figure S2A and B.

### Identifying differentially methylated cytosines (DMCs) commonly presenting in both the parental cell lines and the OxPt-resistant cell lines

In addition to the analysis of the uniquely presenting loci in the parental cell lines (set *P*) or in drug-resistant cell lines (sets *O* and *S*), we extracted the loci that were commonly presented in the OxPt-resistant cell lines and their parental cell lines. DNA methylation entropy analysis [31] was applied to identify the differentially methylated cytosines that presented high diversity of DNA methylation state across all the cell line samples. There were 1,089,634, 2,105,795 and 726,658 cytosine loci identified as OxPt-resistant phenotype associated methylation loci in the context of CpG, CHH and CHG, respectively. We hypothesized that colorectal cancer cells either from *in vitro* samples (the cell line models) or from *in vivo* samples (the clinical samples) share common epigenetic alterations, which are epigenetic changes responsible for the development of an OxPt-resistant phenotype. Thus, we transferred the extracted OxPt-resistant phenotype associated methylation loci from the analysis of the cell line models to the clinical samples.

Because the methylomes of the patient samples represented the DNA methylation profiles prior to drug treatment, we tested whether the identified ‘OxPt resistant phenotype associated methylation loci’ in the cell line models could classify the patients into good or poor outcome groups correctly.

Briefly, we extracted the ‘OxPt resistant phenotype-associated methylation loci’ from the 14 RRBS clinical samples. Subsequently, we performed unsupervised clustering analysis based on the extracted loci to see whether the identified loci in the cell line models could group the patients according to good or poor outcome to treatment (complete response (CR) and partial response (PR) versus no change (NC) and progressive disease (PD)). However, we did not obtain a clearly distinguishable grouping according to good and poor phenotypes (data not shown).

In the next step, we performed a prediction analysis for individual patients to see whether the identified ‘OxPt resistant phenotype associated methylation loci’ could correctly predict the outcome for each patient. We used the DNA methylation information of the identified loci extracted from all the 14 patients as a training data set to select key features and then build a predictor using K-Nearest Neighbour (KNN) [36]. Then, we performed a leave-one-out validation to estimate the accuracy of the prediction. For the ‘OxPt resistant phenotype associated loci’ in the context of CpG, we got an accuracy of 35.7% for the clinical samples to be predicted as good or poor outcome group correctly (Fisher exact test, p-value = 0.59). The OxPt resistant phenotype associated CHH and CHG loci showed 35.7% (p-value = 0.3) and 64.3% (p-value = 0.5804) accuracies, respectively. These results show that it is hard to precisely predict the outcome for individual patients simply based on the DNA methylation state in certain regions identified by the limited number of cell line models. This suggests that DNA methylomes of sporadic clinical samples may show a large diversity in epigenetic reprogramming during the development of drug resistance. The high variability of inter-individual epigenomic profiles poses a big challenge for the selection of useful epigenetic markers for clinical practice.

## Discussion

### Chemotherapeutic agents triggering genotoxic stress

Irinotecan is activated by hydrolysis to SN38 which is a topoisomerase I inhibitor [37]. Inhibition of topoisomerase I by SN38 can result in repression of both DNA replication and transcription [37]. Oxaliplatin is a platinum-based chemotherapeutic agent [38], which exerts its effects by interfering with the DNA replication and transcription machinery through nuclear DNA adduct formation [39]. In the clinic, oxaliplatin’s efficacy depends on combined use with 5-fluorouracil (5-FU) [40]. Capecitabine is a prodrug, that is enzymatically converted to 5-fluorouracil [41], which inhibits the production of nucleotide thymidine by inhibiting the enzyme thymidylate synthase [42]. These chemotherapeutic agents are able to kill the bulk of cancer cells by introducing stress. However, in some cases, stress also can reactivate retrotransposition in somatic cells. For instance, Hagan et al., reported that Alu retrotransposition can be induced by exposure to a variety of genotoxic stressors including the topoisomerase II inhibitor etoposide [19]. In addition, non-genotoxic stress such as hypoxia, contributing to the cancer phenotype including drug resistance and genomic instability, can increase transcription of SINEs (mainly Alu elements) and LINEs by global demethylation [43]. Through the analysis of RRBS data for the cell line models, we found the enrichment of Alu sequences, especially the Alu Y subfamily, in the SN38- and oxaliplatin-resistant cell lines, which provides evidence of reactivation of Alu retrotransposition during the development of drug resistance in colorectal cancer cells. This finding sheds light on the potential role of mobility of Alu elements in colorectal cancer chemotherapeutic resistance by presenting a genomic response to environmental stress.

At the molecular level, cancers are complex diseases attributed to the accumulation of multiple risk factors, from genetic predisposition to environmental factors such as diet, lifestyle and exposure to toxic compounds [44,45]. Epidemiological studies suggest that the environment influences cancer aetiology far more decisively than genetics in many types of cancers [44,45]. DNA methylation, as an important and long-term stable epigenetic mechanism, defines cell fate by maintaining gene expression patterns and stabilizing genetic mobile elements. During development, germ line cells and embryonic stem cells show high cell fate dynamics and activity of mobile genetic elements. Accordingly, DNA methylation also shows dynamic change. In somatic cells, cell fate shows a stable differentiated state and mobile genetic elements present in silent states, partly due to DNA methylation locks. However, DNA methylation, as a reversible chemical modification of DNA sequences can also be changed according to environmental changes involving endogenous or exogenous (bio)chemical molecules. DNA methylation changes could lead to instability of cell fate and reactivation of retrotransposons. At the cell level, somatic cells can become dedifferentiated and heterogeneous, through reshuffling of the genome and remodelling of the epigenome by reactivation of retrotransposons. Through the analysis of DNA methylomes from 421 individuals, ranging in age from 14 to 94 year old, Johansson et al., recently demonstrated that aging at least affects DNA methylation of 29% of investigated sites, of which 60.5% are hypomethylated and 39.6% are hypermethylated [46]. Notably, they also found that a higher fraction of sites in repetitive regions is not affected by the process of aging [46]. This observation suggests that reactivation of Alu retrotransposition presented in our study is not a passive outcome of aging but reflecting a response from mobile genetic elements to environmental stress in line with the finding that the expression of Alu RNAs is shown to increase in response to cellular stress, viral and translational inhibition [8]. Of particular interest is that many prior observations support a correlation between alterations of DNA methylation of retrotransposons and colorectal cancers [20,21,47,48].

### Correlation between Alu retrotransposition and cell stemness

An increasing body of evidence indicates that integration of L1 and Alu elements occurs in germ cells or during early embryonic development [5]. Furthermore, it is reported that the most expressed Alu elements are enriched for the youngest subfamily Y in hESCs [49], which is also in agreement with their recent evolutionary amplification in humans [8]. Additionally, non-CpG methylation has been reported to occur in an asymmetric, strand-specific manner in SINEs and LINEs in hESCs and iPSCs, which is a characteristic property of pluripotent cells [50].

The *SOX1* and other *SOX* gene family members being representative of stemness-related genes were identified loci in set *E*. Furthermore, high diversity of DNA methylation states of *SOX1* and other *SOX* genes presented in the clinical samples suggests that the cancer cells from different patients are variably dedifferentiated. Notably, *SOX1*, *SOX2* and *SOX3* compose the *SOXB1* gene subfamily, which shares more than 90% amino acid identity with respect to the DNA binding high-mobility group (HMG) box (a key characteristic sequence feature for defining *SOX* gene family) and also a high degree of sequence similarity outside the HMG box [51]. Moreover, *SOX1* or *SOX3* can substitute for *SOX2* to produce iPS cells [51]. The *SOXB1* genes are frequently co-expressed in development and exhibit high biological redundancy. In our previous studies, *SOX2* and other *SOX* gene family members were implicated in the development of resistance to the anti-cancer drug tamoxifen [52]. Moreover, the tamoxifen-resistant breast cancer cell lines shared some common features with iPSCs (Li et al., in preparation). By sequencing small cell lung cancers, Rudin et al., reported that a considerable portion of mutations occurring in *SOX2* and other *SOX* genes indicated a correlation between lung cancers and cell stemness [53]. Our observations in the colorectal cancers and in the tamoxifen-resistant breast cell line models ([52]; Li et al., in preparation) also suggest that the *SOX* gene family might play an important role in tumorigenesis and drug resistance.

### Alu retrotransposition increases uncertainty for cancer progression

In the present study, we initially hypothesized that development of drug resistance in colorectal cancer follows a linear step-wise progressive model. In this hypothesis, we assume that all colorectal cancer cells undergo a common path to develop drug resistance, thereby presenting recurrent landmarks of epigenetic alterations. Based on this hypothesis, we should be able to use the selected ‘OxPt resistant phenotype-associated methylation loci’ from the cell line models to predict the outcome of response to oxaliplatin for the clinical samples based on the information of DNA methylation from the primary tumors. However, we could not find such statistically significant predictors to precisely predict the outcome of treatment for the patients. An alternative hypothesis is that drug resistance may follow a non-linear model, in which changes in genomes, epigenomes and cell fate could happen as part of the same mechanism, i.e., retrotransposition, but individual patients could show a diversity of reshaped genome and epigenome and high dynamics of cell fate states because of their different initial conditions and potential stochastic events during the development of drug resistance.

Alus act as endogenous genomic parasites using a ‘copy-and-paste’ mechanism to spread their copies to new locations in the human genome, which can bring three main biological consequences: reshuffling genomes, remodelling epigenomes and reprogramming cell fates. All of these will contribute to heterogeneity of cancers, posing a big challenge for cancer therapy. A given chemotherapy can kill many, even most, of the cancer cells. On the other hand, cancer chemotherapy using certain molecules targeting a given single target or pathway might lose some of their effectiveness if the cancer cells have acquired increased genetic diversity and changed cell fate. In the clinic, one failed therapeutic protocol will be replaced by another one with new chemotherapeutic agent(s). This strategy usually is effective at the beginning, because a new environmental stress is introduced to the cancer cells. However, retrotransposition might act as a genomic response to environmental stress again and eventually lead to resistance to the second treatment as well. If a tumor can be detected at a very early phase, the number of malignant cells is still limited. The probability of development of fitness phenotypes by retrotransposition from the limited number of cells is lower than that from large number of cells in a late phase tumor during treatment. This non-linear model thus fits the clinical notion that ‘earlier detection leads to better outcome’. Additionally, this model also fits the McClintock doctrine, that increases in mobile genetic element transcription that are caused by environmental stress lead to higher levels of mobile genetic element integration and these insertions have an impact on host phenotypes and/or survival [5,33].

## Conclusion

**Figure 7:**
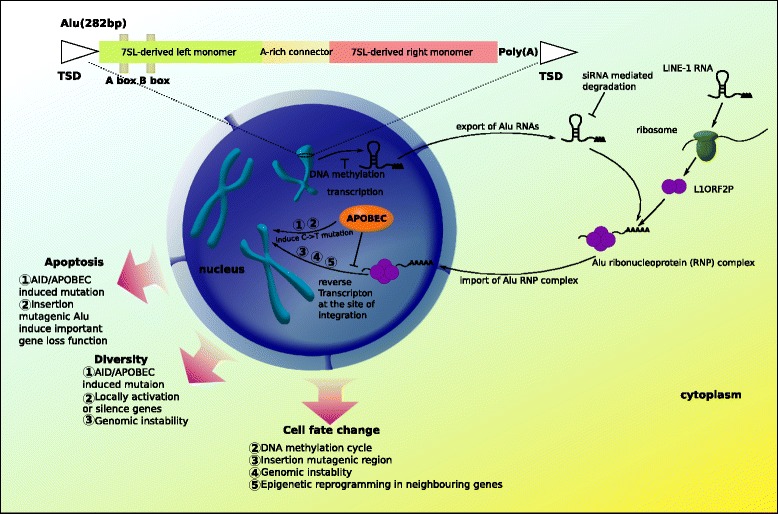
The potential role of Alu mobility in development of drug resistance in colorectal cancer cells. A typical model Alu element is approximately 300 bp in length and has a dimeric structure. The elements are composed of two similar but not-equivalent monomers (7SL-derived left monomer and 7SL-derived right monomer) joined by an A-rich linker. In the 7SL-derived left monomer, there are Box A and Box B, serving as internal Pol III promoter elements, which are helped by an up-stream Pol III enhancer for efficient transcription. The right monomer is followed by a short poly(A) tail and the both terminal sequences typically are Target Site Duplication (TSD) sequences (typically, AA\TTTT). In somatic cells, Alu elements are silent. During tumorigenesis and cancer therapy, in response to the environmental stress induced by carcinogen(s) or chemotherapeutic drug(s), the mobility of Alu elements is activated as a genomic response. Alus propagate using a ‘copy and paste’ mechanism. In the ‘copy’ phase, Alus are typically transcribed by RNA polymerase III. For the ‘paste’ phase, Alus use a ribonucleoprotein complex composed of an endonuclease and a reverse-transcriptase encoded by L1. The endonuclease initially cleaves one DNA strand, and the reverse-transcriptase copies an Alu transcript into a single strand of DNA at that genomic location. The second DNA strand is cleaved by an unknown mechanism, and then the DNA repair mechanism generates the strand complimentary to the novel Alu insertion. The process is named Target-Primed Reverse Transcription (TPRT). Because of the two distinct single-strand breaks, the final DNA sequence contains a TSD, which is a sequence of 4 ~ 25 bp repeated just before and just after the new Alu element.

We have summarized the potential role of Alu elements in colorectal cancer chemotherapy resistance in Figure 7. From this model, one can envision that a single mechanism - reactivation of retrotransposition - will bring an ‘unpredictable’ outcome. Because specificity of target insertion of retrotransposons is weak and individual host genomes and their modifications (epigenomes) are different, spread of Alu copies in the genome will generate a diversity of reshaped genomes and epigenomes. Somatic retrotransposition as genomic response to environmental stress occurs in a discernible but initially unforeseen development [33]. Here, the two features of the non-linear model are dependent on individual genomes and epigenomes (i.e., sensitive dependence of initial condition) and initially in an unforeseen circumstance (i.e., dynamics and evolution). These two features are two key characteristics of complex systems. Therefore, system theory and methodology, applied for interpreting complex systems might be useful for cancer research. In clinical practice, sensitive dependence of initial condition emphasizes the concept of personalized medicine. Following the same simple mechanism (retrotransposition), during tumor development or acquiring resistance to cancer therapy, individual cancers could show different landscapes of genomes and epigenomes. From intensive large scale cancer genome sequencing, even single types of cancers show high diversity and it is quite hard to find individual mutations that can commonly explain all cases. Some genetic or epigenetic features, such as CIMP (CpG island methylator phenotype) [54,55], can match some subtypes of cancers, but every cancer is unique. In addition to the emphasis on individuality, dynamics are important points to focus on in cancer therapy, because mobile genetic elements are not simple genomic parasites but they also contribute to fitness phenotypes of host genomes, fuelling evolution.

Consequently, our study underscores the uniqueness of individual cancers, dynamic tracking of cancer progression and new therapy strategies targeting the entire cell system.

## Methods

### SN38- and OxPt- resistant cell line models

#### Cell culture and generation of drug resistant cell lines

The cell lines HCT116 and HT29 were obtained from the NCI/Development Therapeutics Program, while LoVo was obtained from the American Tissue Culture Collection. Cells were maintained at 37°C, 5% CO2 in RPMI 1640 + Glutamax growth medium (Invitrogen, Naerum, Denmark) supplemented with 10% foetal calf serum (Invitrogen). Oxaliplatin or SN-38 resistant cell lines were generated in our laboratory over a period of 8-10 months by continuous exposure to gradually increasing concentrations of drug [30]. The cell lines were passaged three times at each drug concentration and cell vials were frozen at each increase in drug concentration. Prior to subsequent experiments, the cells were maintained in drug-free growth medium for at least 1 week. The strategy to establish the three drug resistant cell line models is presented in Additional file 8: Figure S3.

The cell line identity of parental and resistant cell lines were confirmed using a short tandem repeat DNA analysis (IdentiCell – Cell Line Authentication Service, Aarhus University Hospital, Aarhus, Denmark). In addition, all cell lines were confirmed to be mycoplasma-free (Mycoplasma PCR Detection Kit, Minerva Biolabs, Berlin, Germany).

#### Chemotherapeutic drugs

Oxaliplatin (Eloxatin, 5 mg/ml, Sanofi-Aventis, Paris, France) was stored at 4°C protected from light. SN-38 (Sigma-Aldrich, Copenhagen, Denmark) was dissolved in dimethyl sulfoxide (DMSO) at a concentration of 10 mM and stored at -20°C. Drugs were diluted in growth medium immediately prior to use.

### The clinical colorectal cancer samples

The timeline of the 14 patients under medical care is shown in Additional file 9: Figure S4. Fresh frozen tumor samples were obtained from a previously published cohort [56], and were collected prior to any chemotherapy. The clinicopathological information from the 14 colorectal cancer patients has been displayed in Additional file 10: Table S6. According to outcome of the therapy (CR and PR versus NC and PD), the 14 patients were divided into a ‘benefited’ and a ‘not benefited’ group (Additional file 10: Table S6). By histological examination, the percentage of tumor cells were evaluated to account for more than 70% (except MOMA5 and MOMA22 with 60% tumor cells) of the cells in each sample. The genomic DNA was isolated from the samples and passed the quality control for construction of RRBS libraries. Written informed consent was obtained from all patients and was approved by The Regional Ethics Committee (DK: 1999/4678).

### RRBS library generation and sequencing

RRBS was performed as previously described [52]. Briefly, 5 μg genome DNA from the cell line models and the clinical samples was digested by restriction enzyme, *MspI* (New England BioLabs) over night at 37°C and QIAGEN Mini Purification kit was used to purify the digested products. End repair was performed, adding A and adaptors in which the cytosines in the paired end adaptor sequence were methylated. The ligated product was subjected to size selection in 2% agarose gel (Bio-RAD) at 100 V for 2 hours. Agarose gel bands with the inserted genomic DNA size 40 ~ 110 bp and the inserted genomic DNA size 110 ~ 220 bp were excised, so that two libraries were generated from each of the MOMA3, MOMA4, MOMA5, MOMA7, MOMA8 and MOMA9 samples (one consisting of 40 ~ 110 bp target sequences and the other of 110 ~ 220 bp target sequences). The rest of the clinical samples and all cell line samples were generated with a single library with inserted DNA fragments of 40 ~ 300 bp length. The DNA from the excised gel pieces was recovered with the QIAGEN Gel Extraction Purification Kit, followed by bisulfite treatment using ZYMO EZ DNA Methylation-Gold kit. The resulting converted DNA was amplified by PCR and purified. The RRBS libraries were subjected to paired-end 50 nt sequencing with HiSeq 2000 (Illumina).

### Bioinformatic analysis

The adaptor sequences were filtered out before the subsequent analysis and the resulting reads were aligned using Bismark software [57]. Only uniquely mapped reads, which had the restriction enzyme cutting site at the 5’ end were used in the subsequent analysis. The sequencing depth and the percentages of methylated cytosines/total investigated cytosines for each C location were calculated. The genomic annotation information was based on the hg19 human genome (http://genome.ucsc.edu). Differentially methylated regions (DMRs) were identified by quantitative differentially methylated regions (QDMR) [31]. The QDMR is a quantitative approach to quantify methylation difference and identify DMRs from genome-wide methylation profiles with a concept of ‘DNA methylation entropy’ [31]. The ‘DNA methylation entropy’ adapting Shannon entropy was used to estimate diversity (or variety) of DNA methylation states for a given locus across samples [31]. We applied WebLogo 3.3 [34] to extract the flanking sequence (up- and down- stream 20 bp) motifs for the investigated genomic sequences. We applied GeneCluster 2.0 [36] to perform the supervised cluster analysis and prediction analysis.

### Availability of supporting data

The data set supporting the results of this article is available in the NCBI Gene Expression Omnibus database accession number (for the raw data and metadata of RRBS for the three colorectal cancer drug-resistant cell line models and the 14 sporadic clinical colorectal cancer samples) is GSE56269. http://www.ncbi.nlm.nih.gov/gds/?term=GSE56269.

## Additional files

Additional file 1: Figure S1. Methylome profiles of the colorectal cancer cell line models and the clinical colorectal cancer samples. Unsupervised clustering profiles of differentially methylated cytosines (DMCs) in the RRBS data for the three colorectal cancer cell line models and the clinical 14 colorectal cancer patients in the context of CpG (Supplementary Fig. 1A), CHG (Supplementary Fig. 1B), and CHH (Supplementary Fig. 1C). The DNA methylation level is shown as percentage. Full green color means 100 percent DNA methylation, whereas full red color means 0 percent DNA methylation. The intermediate DNA methylation levels are shown in gradient color between full green and full red according to the DNA methylation level (percentage). Additional file 2: Table S1. The information of non-CpG cytosine methylation (CHG in Supplementary Table 1A and CHH in Supplementary Table 1B) in the colorectal cancer cell line models and the sporadic colorectal cancer samples. Additional file 3: Table S2. The definitions of the sets in this study. Additional file 4: Table S3. Information on the identified cytosine loci in set *E*. Additional file 5: Table S4. Information on variance of the percentage of Alu Y in Alu elements in all RRBS samples and variance of the percentage of Alu Y in Alu elements in the simulations. Additional file 6: Table S5. List of involved genes and the number of their harboured cytosine loci identified in set *E*. Additional file 7: Figure S2. The DNA methylation state (Supplementary Fig. 2A) and the diversity of DNA methylation (entropy) (Supplementary Fig. 2B) of the *TSPYL2* gene between the 14 clinical sample. Additional file 8: Figure S3. The strategy of establishing the three drug-resistant cell line models. Additional file 9: Figure S4. The timeline of the 14 colorectal patients in medical treatment. Additional file 10: Table S6. The clinicopathological information of the 14 sporadic colorectal cancer patients.
