# Supplementary material for: The potential role of Alu Y in the development of resistance to SN38 (Irinotecan) or oxaliplatin in colorectal cancer
Source: BMC Genomics. 2015 May 22;16(1):404. doi: 10.1186/s12864-015-1552-y (PMC4440512; doi:10.1186/s12864-015-1552-y)
Supplement: Additional file 1: Figure S1. — Methylome profiles of the colorectal cancer cell line models and the clinical colorectal cancer samples. Unsupervised clustering profiles of differentially methylated cytosines (DMCs) in the RRBS data for the three colorectal cancer cell line models and the clinical 14 colorectal cancer patients in the context of CpG (Supplementary Fig. 1A), CHG (Supplementary Fig. 1B), and CHH (Supplementary Fig. 1C). The DNA methylation level is shown as percentage. Full green color means 100 percent DNA methylation, whereas full red color means 0 percent DNA methylation. The intermediate DNA methylation levels are shown in gradient color between full green and full red according to the DNA methylation level (percentage). [file 12864_2015_1552_MOESM1_ESM.zip › 1709793881135619_add1.pdf]

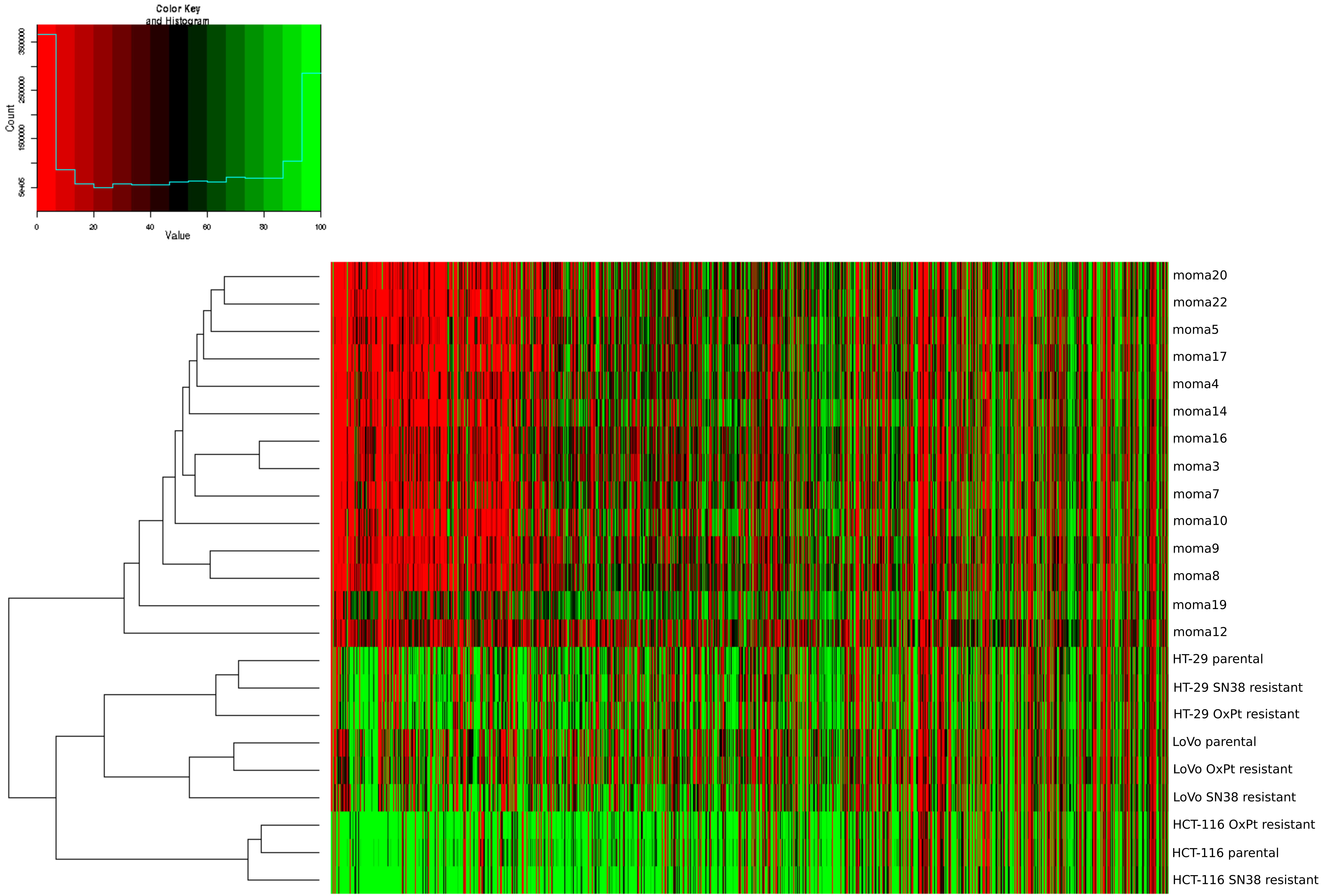
Color Key
and Histogram
Count
3500000
2500000
1500000
5e+05
0
20
40
60
80
100
Value
moma20
moma22
moma5
moma17
moma4
moma14
moma16
moma3
moma7
moma10
moma9
moma8
moma19
moma12
HT-29 parental
HT-29 SN38 resistant
HT-29 OxPt resistant
LoVo parental
LoVo OxPt resistant
LoVo SN38 resistant
HCT-116 OxPt resistant
HCT-116 parental
HCT-116 SN38 resistant
