# Supplementary figures and images for: The potential role of Alu Y in the development of resistance to SN38 (Irinotecan) or oxaliplatin in colorectal cancer
Source: BMC Genomics. 2015 May 22;16(1):404. doi: 10.1186/s12864-015-1552-y (PMC4440512; doi:10.1186/s12864-015-1552-y)

Color Key  
and Histogram

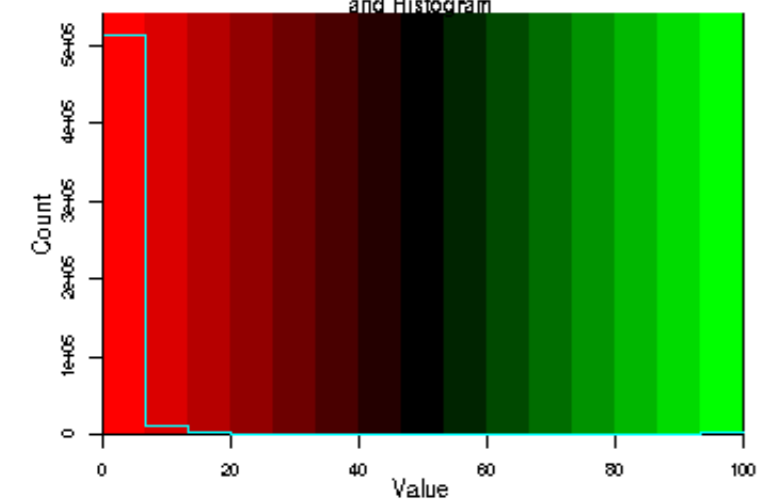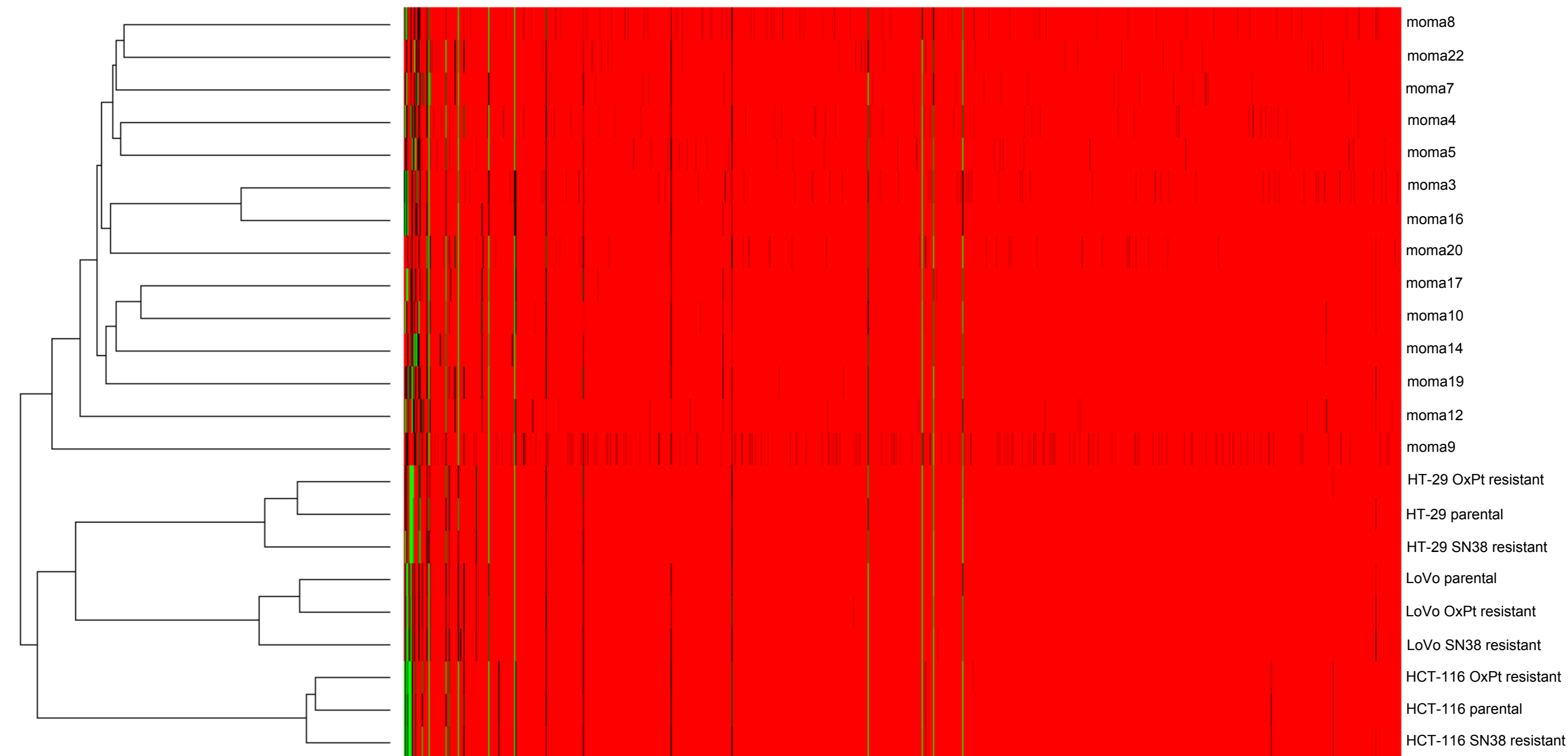

Supplement: Additional file 1: Figure S1. — Methylome profiles of the colorectal cancer cell line models and the clinical colorectal cancer samples. Unsupervised clustering profiles of differentially methylated cytosines (DMCs) in the RRBS data for the three colorectal cancer cell line models and the clinical 14 colorectal cancer patients in the context of CpG (Supplementary Fig. 1A), CHG (Supplementary Fig. 1B), and CHH (Supplementary Fig. 1C). The DNA methylation level is shown as percentage. Full green color means 100 percent DNA methylation, whereas full red color means 0 percent DNA methylation. The intermediate DNA methylation levels are shown in gradient color between full green and full red according to the DNA methylation level (percentage). [file 12864_2015_1552_MOESM1_ESM.zip › 1709793881135619_add2.pdf]

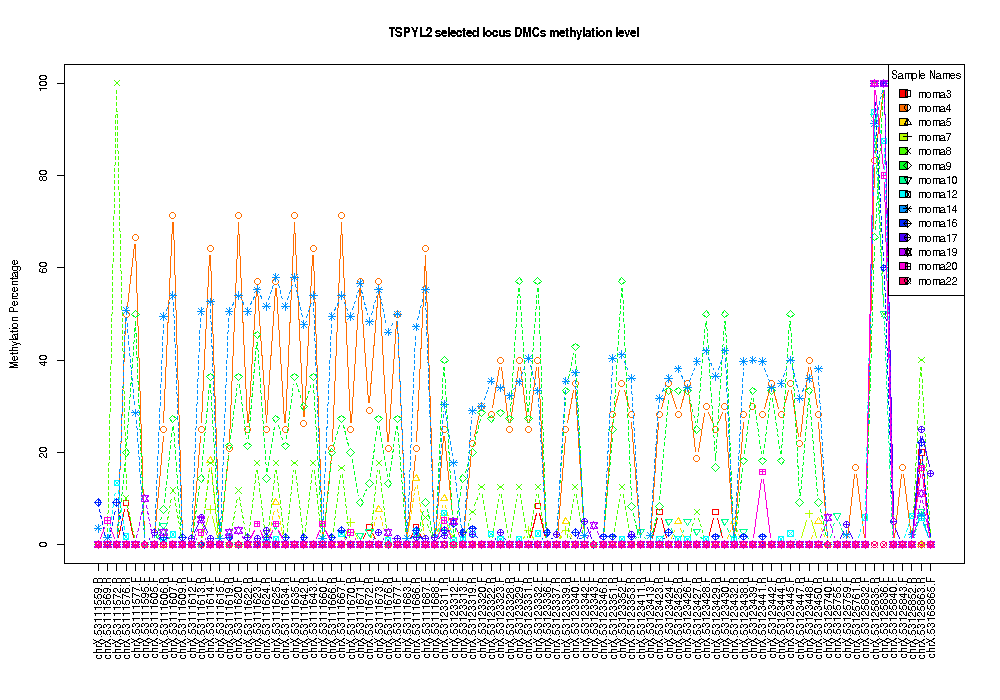

Supplement: Additional file 7: Figure S2. — The DNA methylation state (Supplementary Fig. 2A) and the diversity of DNA methylation (entropy) (Supplementary Fig. 2B) of the TSPYL2 gene between the 14 clinical sample. [file 12864_2015_1552_MOESM7_ESM.zip › 1709793881135619_add4.png]

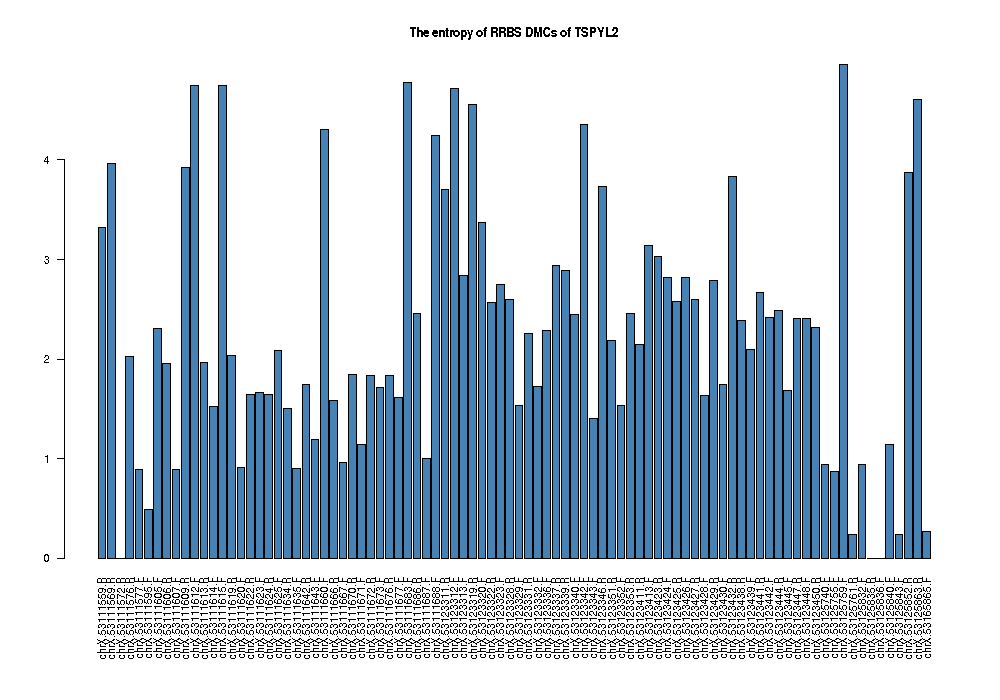

Supplement: Additional file 7: Figure S2. — The DNA methylation state (Supplementary Fig. 2A) and the diversity of DNA methylation (entropy) (Supplementary Fig. 2B) of the TSPYL2 gene between the 14 clinical sample. [file 12864_2015_1552_MOESM7_ESM.zip › 1709793881135619_add5.png]
