# Supplementary material for: The potential role of Alu Y in the development of resistance to SN38 (Irinotecan) or oxaliplatin in colorectal cancer
Source: BMC Genomics. 2015 May 22;16(1):404. doi: 10.1186/s12864-015-1552-y (PMC4440512; doi:10.1186/s12864-015-1552-y)
Supplement: Additional file 3: Table S2. — The definitions of the sets in this study. [file 12864_2015_1552_MOESM3_ESM.docx]

***Set P***: the cytosine loci uniquely presented in the three parental cell lines (the cell line A (HCT-116 parental), D (HT-29 parental) and G (LoVo parental)) as unique drug-sensitive DNA methylation features, in the three formats (CpG, CHG and CHH).

***Set O***: the cytosine loci uniquely presented in the three OxPt-resistant offspring cell lines (the cell line B (HCT-116 OxPt resistant), E (HT-29 OxPt resistant) and H (LoVo OxPt resistant)) as unique OxPt-resistant DNA methylation features, in the three formats (CpG, CHG and CHH).

***Set S***: the cytosine loci uniquely presented in the three SN38-resistant offspring cell lines (the cell line C (HCT-116 SN38 resistant), F (HT-29 SN38 resistant) and I (LoVo SN38 resistant)) as SN38-resistant DNA methylation features, in the three formats (CpG, CHG and CHH).

***Set C***: the cytosine loci shared by all the 14 clinical samples, in the three formats (CpG, CHG and CHH).

***Set E***: the cytosine loci commonly presented in the set *C* and the united set of *P*, *O* and *S*.

***Set A***: the common cytosine loci shared by all three cell line models (total the nine colon cancer cell lines).
