# Supplementary material for: The potential role of Alu Y in the development of resistance to SN38 (Irinotecan) or oxaliplatin in colorectal cancer
Source: BMC Genomics. 2015 May 22;16(1):404. doi: 10.1186/s12864-015-1552-y (PMC4440512; doi:10.1186/s12864-015-1552-y)
Supplement: Additional file 8: Figure S3. — The strategy of establishing the three drug-resistant cell line models. [file 12864_2015_1552_MOESM8_ESM.pptx]

## Slide 1
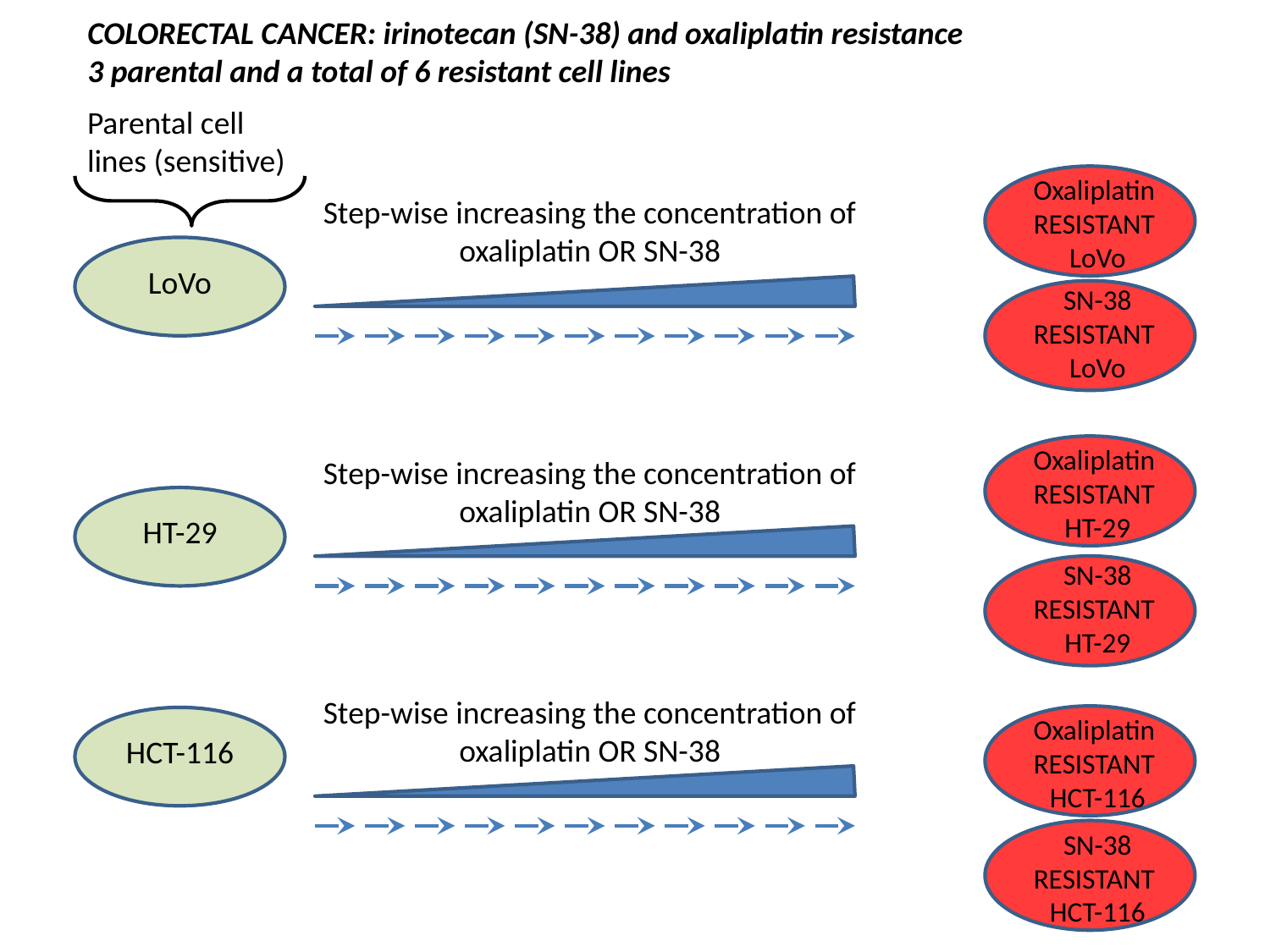

COLORECTAL CANCER: irinotecan (SN-38) and oxaliplatin resistance
3 parental and a total of 6 resistant cell lines
Parental cell lines (sensitive)
Oxaliplatin
RESISTANT
LoVo
Step-wise increasing the concentration of oxaliplatin OR SN-38
LoVo
SN-38
RESISTANT
LoVo
Oxaliplatin
RESISTANT
HT-29
Step-wise increasing the concentration of oxaliplatin OR SN-38
HT-29
SN-38
RESISTANT
HT-29
Step-wise increasing the concentration of oxaliplatin OR SN-38
Oxaliplatin
RESISTANT
HCT-116
HCT-116
SN-38
RESISTANT
HCT-116
