# Supplementary material for: The potential role of Alu Y in the development of resistance to SN38 (Irinotecan) or oxaliplatin in colorectal cancer
Source: BMC Genomics. 2015 May 22;16(1):404. doi: 10.1186/s12864-015-1552-y (PMC4440512; doi:10.1186/s12864-015-1552-y)
Supplement: Additional file 9: Figure S4. — The timeline of the 14 colorectal patients in medical treatment. [file 12864_2015_1552_MOESM9_ESM.pptx]

## Slide 1
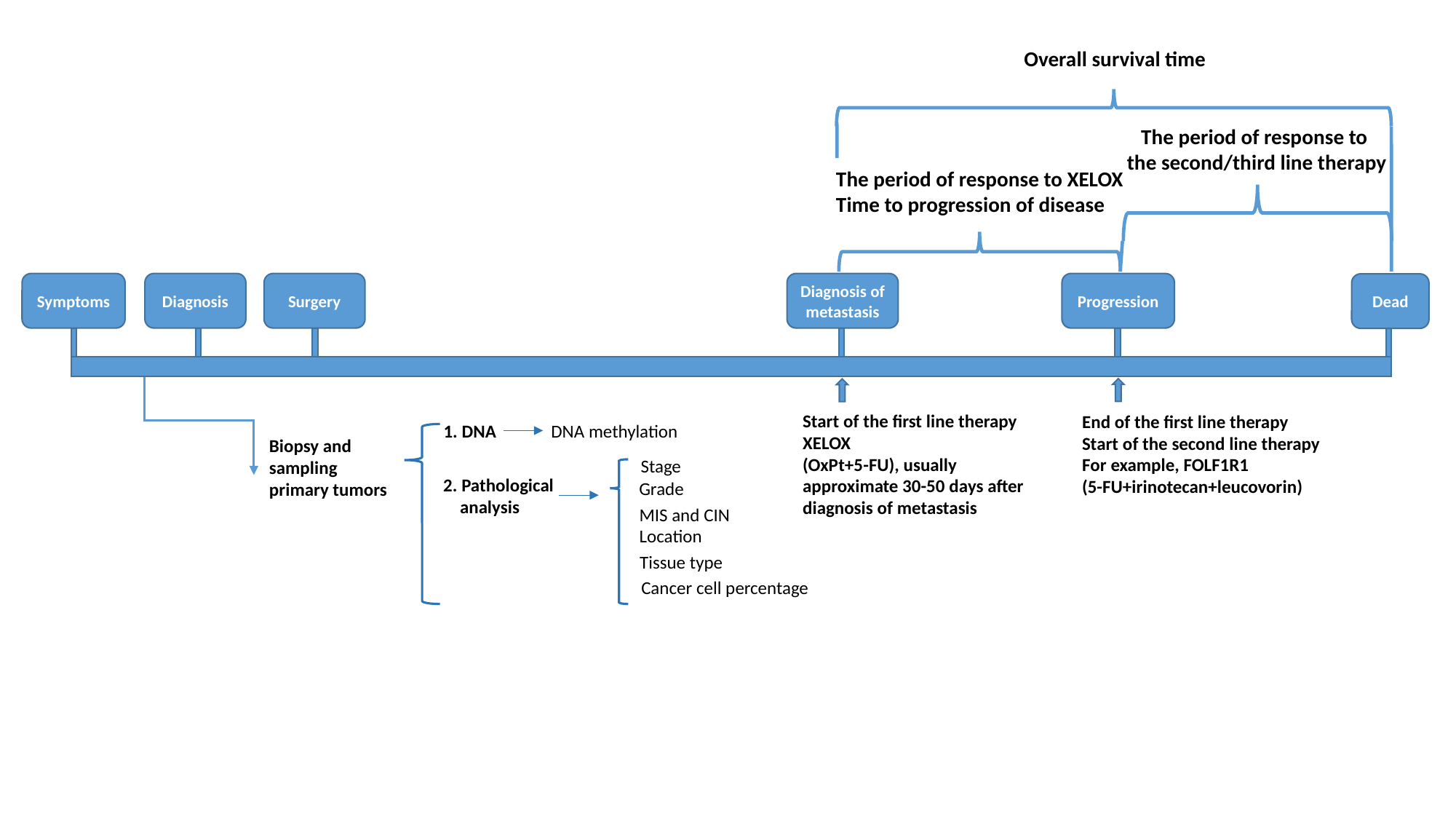

Overall survival time
The period of response to
the second/third line therapy
The period of response to XELOX
Time to progression of disease
Diagnosis
Diagnosis of metastasis
Progression
Symptoms
Surgery
Dead
Start of the first line therapy
XELOX
(OxPt+5-FU), usually approximate 30-50 days after diagnosis of metastasis
End of the first line therapy
Start of the second line therapy
For example, FOLF1R1
(5-FU+irinotecan+leucovorin)
DNA methylation
1. DNA
Biopsy and sampling primary tumors
Stage
2. Pathological
 analysis
Grade
MIS and CIN
Location
Tissue type
Cancer cell percentage
